# Advances in Transcription Factors Related to Neuroglial Cell Reprogramming

**DOI:** 10.1515/tnsci-2020-0004

**Published:** 2020-02-20

**Authors:** Kuangpin Liu, Wei Ma, Chunyan Li, Junjun Li, Xingkui Zhang, Jie Liu, Wei Liu, Zheng Wu, Chenghao Zang, Yu Liang, Jianhui Guo, Liyan Li

**Affiliations:** 1 Institute of Neuroscience, Kunming Medical University, Kunming, Yunnan, China; 2 Second Department of General Surgery, First People’s Hospital of Yunnan Province, Kunming, Yunnan, China

**Keywords:** Neuroglial cell, Reprogramming, Transcription factor

## Abstract

Neuroglial cells have a high level of plasticity, and many types of these cells are present in the nervous system. Neuroglial cells provide diverse therapeutic targets for neurological diseases and injury repair. Cell reprogramming technology provides an efficient pathway for cell transformation during neural regeneration, while transcription factor-mediated reprogramming can facilitate the understanding of how neuroglial cells mature into functional neurons and promote neurological function recovery.

## 1 Introduction

Neuroglial cells are non-neuronal cells and have received great attention because glial cells have been regarded as important modulators of many aspects of brain function and disease [1]. The term “neuroglia” was originally proposed by Virchow in 1850, who assumed that neuroglia were a class of cells that consisted of neurons embedded in a layer of connective tissue, referred to as “glia”. Glial cells in the CNS consist of astrocytes, oligodendrocytes and microglia, while glial cells in the peripheral nervous system (PNS) consist of Schwann cells (SCs) and satellite glia. Neuroglial cells are intimate partners of neurons throughout their life cycle [2]. In embryos, neuroglial cells form a cellular framework and regulate the survival and differentiation of neurons. In addition, during neurogenesis and early development, neuroglial cells mediate the proliferation and differentiation of neurons by synthesizing and secreting various growth factors and extracellular matrix components [2]. The most prominent function of neuroglial cells during development is formation of myelin sheaths around axons, which provide necessary signals and maintain rapid conduction for nervous system function [3]. Additionally, neuroglial cells maintain homeostasis in nerve cells and participate in synaptic plasticity and cell repair [2]. Similar to developmental processes in other types of animal cells, the development of neuroglial cells is influenced by interactions between cells; cell lineage and extracellular signaling can regulate the migration, proliferation and differentiation of glial cells. In recent years, by isolating different types of glial cells for culture and in vitro growth studies, researchers have made substantial progress in identifying the types of microglial cells and factors that affect the development of neuroglial cells [4]. Thus, the application of cell reprogramming technology has become a focus of research. Neuroglial cell reprogramming can be mediated by cytokines, epigenetic factors and transcription factors. DNA methylation and proteomics also play key regulatory roles in this process, and cell reprogramming technology is widely used to examine the roles of these factors. This review focuses on the research progress in examining the regulation of neuroglial cell reprogramming by transcription factors (Table 1).

**Table 1:** Transcription factors regulate glial cell reprogramming

Cell Types	Related Transcription Factors	Cell Generated (other nerve regeneration)	References
**Central Nervous System**			
Astrocyte	NeuroD1	Neuron	[5]
Astrocyte	SOX2	DCX+ Neuron	[19]
Astrocyte	ASCL1, Neurog2	Neuron	[23]
Astrocyte	DLX2	GABA Neuron	[42]
Astrocyte	Neurog2	Glutamatergic Neuron	[42]
NG2 glial cell	SOX2	DCX + Neuron	[29]
Static astrocyte	SOX2	Neuroblast	[45]
Reactive astrocyte	PAX6	Neurogenic Cell	[42]
Reactive astrocyte	NeuroD1	Glutamatergic Neuron	[44]
Oligodendrocyte progenitor cell	SOX2	Nerve-like Stem Cell	[46]
Microglial cells	SOX2	Neural Stem Cell /Progenitor Cell	[47]
**Peripheral Nervous system**			
Schwann cell	C-JUN	Myelination	[53]
Schwann cell	RUNX2	Myelination	[52]
Schwann cell	NF*-κB*	Myelination and Axon Regeneration	[60]
Schwann Precursor Cell	NOTCH	Myelination	[60]
Satellite glial cell	SOX10, MYRF, NKx2.2	Oligodendrocyte-like Cell	[68,69]

## 2 Definition of neuroglial cell reprogramming

In the nervous system, all methods of transforming non-neuronal cells into neurons are presently caused damage to brain, and the emergence of cell reprogramming technology may allow non-neuronal cells to produce a variety of specific cell types, including neurons [5]. In cell reprogramming, direct reprogramming, also known as transdifferentiation, can transform one somatic cell type directly into another without inducing pluripotency. Cell reprogramming can be implemented using many methods, each of which has its own advantages and disadvantages. The reprogramming process typically uses regulatory factors to improve cell characteristics and mediate functional development [6]. Generally, three main approaches are used. First, exogenous transgenes can be introduced into cells to overexpress key transcription factors and initiate the process of transdifferentiation [7, 8, 9, 10]. Second, direct regulation of DNA or epigenetics methods, such as CRISPR/Cas9 gene editing, can specifically target, silence or up-regulate endogenous genes that are critical for the process of transdifferentiation [11, 12, 13, 14]. Finally, drug-targeted transcription factors can be used to induce a cellular immune response [15], which then induces a cascade effect and epigenetic remodeling or directly changes the epigenetic environment [16, 17]. In recent years, direct reprogramming of neuroglial cells has been achieved by constructing vectors that overexpress transcription factors, which have been used for small molecule research and CRISPR/Cas9 gene therapy. Lentiviral vectors overexpressing transcription factors are the most popular technology at present [6]. Brulet et al [5] proposed that NEUROD1, a non-invasive vascular transdifferentiation factor, can be used to produce new neurons. They used adenovirus AAV9 to deliver NEUROD1 to astrocytes via intravascular pathways, and a small fraction of non-reactive astrocytes in the striatum were found to be transformed into neurons, while no astrocytes in the cortex were transformed. These results show that under physiological conditions, a single transcription factor can induce astrocytes to transform into neurons. Even in the absence of reactive glial proliferation, NEUROD1 can also transform astrocytes into neurons. Additionally, after regression of reactive glial proliferation, transcription factors can also mediate the transformation of astrocytes into neurons, which may be helpful for treatment in emergency situations. Furthermore, longterm clinical studies have been conducted in patients after nervous system injury.

Neuroglial cells play a variety of roles in the physiological and pathological processes of the central nervous system, such as maintaining homeostasis, providing neurotrophic proteins to neurons and regulating nerve signaling. Recently, there has been increasing evidence that glial cells can also act as nerve stem/ progenitor cells and contribute to adult neurogenesis or nerve regeneration. For example, astrocytes and oligodendrocyte precursor cells may be activated to proliferate and differentiate under pathological conditions. When cultured in vitro, they can form neural spheres capable of differentiating into astrocytes, oligodendrocytes and neurons. In addition, forced expression of exogenous genes in astrocytes and NG2-glia successfully reprogrammed them into neurons, which may also indicate their stem/progenitor characteristics [18]. Niu’s team [19] screened more than a dozen neural stem cell (NSC) regulators, which play a key role in neurogenesis and cell reprogramming, and found that the stem cell factor SOX2 can produce DCX-positive cells (possibly neuroblasts in the brains of adult mice. SOX2 has also been found to have powerful reprogramming capabilities [20, 21] and in vivo reprogramming of astrocytes depends on SOX2 [22]. Su et al [23] demonstrated that SOX2 could reprogram astrocytes to transform into mature neurons after spinal cord injury in adult mice. These transformed neurons can form synaptic connections with local motor neurons. In summary, SOX2 overexpression initiates a progressive reprogramming process that transforms astrocytes into neuronal progenitor cells and ultimately generates mature neurons in the damaged adult CNS. The multistep reprogramming process that drives SOX2 may provide much-needed neurons for nerve regeneration after injury or degeneration. Studies have also demonstrated that astrocytes can be reprogrammed to transform in vitro into fully functional neurons using a retroviral transcription factor carrying ASCL1 or NEUROG2 [24, 25]. In addition, the co-expression of SOX2 and ASCL1 can induce astrocytes isolated from the adult mouse brain to transform into neurons [26]. When the adult mouse cortex suffers local damage, glial cells proliferate [21, 27]. Three days after injury, these proliferated neuroglial cells could be used in induction experiments targeting SOX2 or ASCL1, and expression of DCX could be observed in the injured tissues. No neurogenesis was observed, but on the seventh day after the experiment, SOX2 alone could induce a large number of DCX-positive cells. Identification of these DCX-positive cells in Sax10- iCreERT2 mice (transgenic mice) revealed that most originated from proliferative NG2 glial cells [28, 29]. Patch clamp recording further confirmed the presence of low-frequency signals in these induced neurons. Although these results were consistent with those of neurons, Bergles et al [30] indicated that some of the signaling characteristics might originate from NG2 glial progenitor cells. In fact, even overexpression of SOX2 could not transform NG2 glial cells or astrocytes into DCX-positive cells without previous cortical tissue damage [31].

Thus, direct reprogramming is caused by different transcription factors in specific cell lines and epigenetic backgrounds [32].

## 3 Transcription factors mediate neuroglial cell reprogramming in the central nervous system

Astrocytes exhibit two states: static and reactive. These states differ in that static astrocytes cannot divide but can proliferate and differentiate when activated by injury states [33] or other pathological conditions such as stroke [34] and neurodegenerative diseases [35]. Activated astrocytes under pathological conditions are reactive and have the potential for neurogenesis, and their abilities lie between those of radial glial cells and static astrocyte [36]. In fact, one study showed that astrocytes produce multilineage precursor cells and NSCs during the early postnatal period [37]. Some reports also indicate that reactive astrocytes in the damaged brain have significant plasticity and the potential to become NSCs. These activated astrocytes can self-renew and be reprogrammed into neurons, astrocytes and oligodendrocytes in vitro [38, 39, 40, 41]. Astrocytes can also produce neurons or NSCs when appropriate transcriptional signals are provided in vitro. Recombinant Pax6 adenovirus can promote the transformation of astrocytes into neurogenic cells in the early postnatal cerebral cortex [24, 25, 42, 43]. However, there is no evidence of the function of these neurons and no indication of their cell type. Therefore, Berninger et al [24] studied the physiological characteristics of neurons derived from these transformed astrocytes. Using the transcription factors NEUROG2 and MASH1, they found that in the early postnatal cortex, astrocytes could be induced to transform into neurons. Spontaneous synaptic activity was not observed during the incubation period. However, when these astrocytes were co-cultured with cortical neurons, functional synaptic signals appeared in the derived neurons, indicating that spontaneous or induced synaptic activity was regulated by the transcription factors NEUROG2 and MASH1. Therefore, it was suggested that astrocytes could be regulated by specific transcription factors, and the derived neurons could facilitate repair of damaged neuronal networks. In addition, Heinrich et al [25] established functional synapses by expressing neurogenic factors, confirming that DLX2 mediates the transformation of postnatal cortical astrocytes in mice into GABAergic neurons, while NEUROG2 was associated with transformation of astrocytes into glutamatergic neurons. Similarly, in addition to transforming astrocytes of postnatal mice, NEUROG2 or DLX2 can also mediate the progressive development of reactive astrocytes into fully functional neurons that can establish functional connections with damaged cerebral cortex astrocytes. These results suggest that endogenous astroglia play an important role in the regeneration of the CNS after brain injury or during pathological states.

Neurogenic factors and REST-assisted inhibitors (neuronal phenotypic regulators) regulate the transcriptional mechanism of astrocyte reprogramming into neurons. Masserdotti et al [32] found that NEUROG2 and ASCL1 mediated early transcription during the process of direct reprogramming of postnatal astrocytes into different neuronal subtypes in vitro. Their study revealed the transcriptional events that occur during the initial stage of astrocyte reprogramming into neurons; this transformation occurs rapidly in a dynamic manner, and ASCL1 and NEUROG2 exhibit a unique transcriptional program. By analyzing the identified target genes, NEUROD4 was shown to act on downstream target genes and affect the direct reprogramming of astrocytes into functional neurons. The combination of REST and the NEUROD4 promoter prevented the recruitment of NEUROG2. Therefore, this process may reveal some of the mechanisms of early inhibition of reprogramming of astrocytes to transform into neurons.

Zhao et al [44] suggested that the transcription factor NEUROD1 could reprogram reactive astrocytes in injury or Alzheimer’s disease (AD) models to transform into glutamatergic neurons, and repetitive action potentials and spontaneous synaptic activity could be detected in these neurons, suggesting that the transformed neurons establish functional connections with peripheral neurons.

In addition to demonstrating that reactive astrocytes or astrocytes in the brain of postnatal mice can be directly reprogrammed into neurons or stem cell-like cells by overexpression of certain transcription factors, induced neuronal stem cells (iNSCs) can also be derived from astrocytes in vivo. Huang and colleagues [45] demonstrated that astrocytes can be directly transformed into neuroblasts. Unlike previous studies, they found that most of the induced neuroblasts originated from stationary astrocytes, suggesting that static astrocytes also exhibit plasticity in vivo. They reprogrammed stationary astrocytes into induced adult neuroblasts (iANBs) using a single transcription factor, SOX2. They also found that brain-derived neurotrophic factors and valproic acid, a cephalin and histone deacetylase inhibitor, promoted iANBs to differentiate into mature neurons, suggesting that the microenvironment is also essential for cell reprogramming. Differentiated neurons have electrophysiological functions; therefore, they can be integrated into local neuronal circuits. In another study, SOX2 was also used to transform static astrocytes into neuroblasts in adult mice with spinal cord injuries.

Oligodendrocyte precursor cells (OPCs) can develop into multipotent neuron-like stem cells (NSLCs), which can self-renew and produce neurons, astrocytes and oligodendrocytes when exposed to certain extracellular signals. This was an important finding in developmental neurobiology, suggesting that glia have the potential to be reprogrammed into stem cells for treatment of neurological diseases [46]. External or intrinsic signals that endow these neuroglial cells with neurogenic potential are associated with four transcription factors (OCT4, SOX2, KLF4 and C-MYC) that may directly reprogram fibroblasts into induced pluripotent stem cells, and these core transcription networks may regulate a neuroglial population [47, 48]. SOX2, as a high mobility group transcription factor, maintains the pluripotency of NSCs [49, 50]. SOX2 is expressed in developing nerve canals and proliferating CNS progenitor cells. The transition from OPCs to NSLCs has been shown to be mediated by SOX2 activation, and this core transcription factor plays a key role in identifying and maintaining the development of NSCs. The BMP signaling pathway is an important modality used to induce reprogramming of OPCs into pluripotent NSCs. Immunocytochemical analysis shows that the BMP signaling pathway regulates SOX2 expression, mediates reprogramming of microglial cells (MGs) and OPCs and up- regulates markers of NSCs such as CD15 and nestin. Protein imprinting and double immunostaining further confirmed that activation of the BMP signal by SOX2 binding may be an important molecular pathway involved in the reprogramming of OPC lineage. This study confirmed that OPCs and MGs could be reprogrammed in vitro, and the reprogrammed cells could exhibit some markers of NSCs or progenitor cells when SOX2 and BMP signal transduction are up-regulated. The mechanisms involved in these processes may help to regulate the fate of neuroglial cells and provide a valuable treatment for neuron loss during neurological diseases [46, 51].

## 4 Transcription factors mediate neuroglial cell reprogramming in the peripheral nervous system.

Myelination of the PNS is essential for maintaining axonal function. After peripheral nerve injury, the function of SCs changes from myelination to demyelination. SC reprogramming affects the survival and demyelination of neurons and their supporting axons. This reprogramming is regulated by distal regulatory elements, which also integrate multiple transcription factors [52].

SCs can repair injured neurons, facilitate their survival and provide necessary pathways for axon regeneration and target innervation. The transformation of reparative SCs involves dedifferentiation and alternative differentiation or activation, commonly referred to as (direct or pedigree) reprogramming. Damage-induced SC reprogramming involves activation of myelin genes and a series of repair factors, including up- regulation of nutrient factors, elevation of cytokines involved in the immune response, activation of the myelin sheath to halt myelin autophagy and macrophage aggregation in SCs and determination of the regeneration trajectory of the target through axon orientation. The repair process involves the regulatory mechanism of the transcription factor c-Jun. After injury, c-Jun is rapidly up-regulated in SCs. In contrast, in the absence of c-Jun, injury leads to dysfunction, neuronal death and functional recovery failure. Therefore, although c-Jun is not necessary for SC development, it is essential for SC reprogramming and post-injury repair [53]. c-Jun levels are low or non-existent in Schwann cell precursors (SCPs) and are elevated in immature SCs, but c-Jun is inhibited during postnatal development. It can be detected in many non-myelinated SCs and in a small number of myelinated cells and adult developing nerves [54, 55, 56, 57, 58]. c-Jun can also control the response of SCs to injury, dedifferentiation of myelin cells and activation of repair programs [56, 59]. The transcription factors NOTCH, SOX2, PAX3 and ID2 can act as negative regulators of myelin formation [54, 55], while c-Jun regulates the myelin gene to inhibit the expression of these negative regulators after injury [53]. Although c-Jun plays an important role in the response of SCs to nerve injury, it also activates other transcription factors, some of which depend on c-Jun, such as RUNX2, which acts as an enhancer that can bind to c-Jun after nerve injury.RUNX2 is also a transcription factor that is necessary to induce the expression of other genes [52].

In SC dedifferentiation, negative regulators of myelin formation can offset the positive regulators of myelin formation in most cases and may play a major role after nerve injury. For example, the transcription regulators SOX2, Pax3, ID2, EGR1 and EGR3 can mediate SC dedifferentiation in damaged nerves. In vitro, overexpression of SOX2, Pax3 and ID2 can reduce the expression of the myelin gene [60]. In addition, SOX2 can help repair the damaged myelin sheath, and this action is mediated by Ephrin-B/EphB2 signaling between fibroblasts and SCs [61].

NOTCH is a transmembrane receptor protein that is lysed after ligand binding to produce an intracellular domain. As a transcriptional regulator, NOTCH controls the proliferation of SCs and promotes the production of immature SCs by SCPs in vivo, but also acts as a negative regulator of myelin formation. During the formation of the myelin sheath, the NOTCH level gradually decreases, and its inactivation or over- activation will lead to premature or delayed myelin sheath formation, respectively [61]. ZEB2 controls SC transdifferentiation and myelin regeneration by recruiting Histone deacetylases 1 and 2 (HDAC1/2) and NURD complexes and inhibiting NOTCH-Hey2 signaling [62]. Nuclear factor-kappa B (NF-kappa B) is a transcription factor that regulates many physiological processes and mediates inflammatory responses in many diseases. Several studies have shown that the activation of NF-kappa B is essential for transformation, axon regeneration and demyelination of SCs in vitro [60].

Furthermore, the binding sites of the transcription factors SOX10 and EGR2/KROX20 cannot be identified by the activity enhancer recognition marker h3k27ac, which is the key determinant of SC differentiation. The expression of the transcription factor EGR2 disappears after nerve injury. h3k27a can accurately indicate the dynamic changes in SC transcription, and it can be used to monitor the effect of transcription factors on myelin formation in SCs and the response of the PNS to nerve injury [52].

Satellite glial cells are a group of cells around primary sensory neurons that are similar to astrocytes in the central nervous system [63]. As a type of peripheral glial cells, satellite glial cells can regulate the neuron microenvironment and signal transmission in sensory ganglia [64]. When satellite glial cells are cultured in vitro, they show plasticity and produce various types of glial cells [65, 66]. Matthias et al [32] found that satellite glial cells can be reprogrammed to some extent, which may be related to their characteristics and the plasticity of their precursors. When SOX10 is overexpressed, a type of elliptic glial cell (possibly satellite glial cells but not SCs) in the peripheral system, which are not SCs, in the PNS tends to transform into oligodendrocyte-like cells. In this microenvironment, satellite glial cells, can closely contact neurons and may provide signal guidance for the development of oligodendrocytes. The expression of endogenous SOX10 in satellite glial cells is important and may be activated when satellite glial cells transform into oligodendrocyte-like cells [66, 67]. SOX10-induced reprogramming of satellite glial cells into oligodendrocytes is also associated with many transcription factors, such as NKx2.2 and MYRF [68, 69].

## 5 Advances in neuroglial cell reprogramming in neurological diseases

A study published in the Journal Nature Biotechnology showed that reprogramming astrocytes into dopaminergic neurons improves motor function in mice with Parkinson’s disease (PD). This method can be used in place of traditional cell replacement. In mice with unilateral striatal astrocyte deletion (a PD model), lentiviral injection induced the overexpression of NeAL218 (via a mixture of three transcription factors, including NEUROD1, ASCL1, LMX1A and microRNA218). At 5 weeks after lentiviral induction of NeAL218, dopaminergic neurons were increased, and motor function had recovered. This in situ reprogramming of astrocytes for PD provides a new possibility for cell replacement therapy, thus avoiding unnecessary cell transplantation or immunosuppression [70]. Rivetti [71] also used NeAL218 to reprogram human astrocytes in vitro and mouse astrocytes in vivo to induced dopamine neurons, further by promoting chromatin remodeling and activating TGFβ, Shh, and Wnt signaling pathways to improve the efficiency of in vitro reprogramming. They found that human astrocytes reprogrammed as efficiently as 16%, resulting in excitatory induced dopamine neurons and improved gait disorders.

Reactive glial cells produced after brain injury in mice or in AD models can reprogram functional neurons directly in vivo through the transcription factor NEUROD1. NEUROD1 mediates reprogramming of astrocytes into glutamatergic neurons and the reprogramming of NG2 glial cells into glutamatergic neurons and GABAergic neurons. This process of reprogramming glial cells into different types of neurons using the same transcription factor may provide important clues to the lineage relationship between neurons and glial cells. Reactive glial cells can be reprogrammed as functional neurons in the brain of injured or diseased mice, which may provide a treatment for reactive gliosis, which is widely associated with nerve injury and neurodegenerative diseases. In situ reprogramming of functional neurons by reactive astrocytes and NG2 cells may provide new research topics for brain repair via internal reprogramming of neurons [72]. However, whether non-viral methods or small molecule strategies can be used to influence reprogramming in vivo has become a major concern of researchers [72, 73, 74]. Another equally challenging question is whether in vivo reprogramming can be used to treat behavioral deficits, such as cognitive impairments, that occur in neurological diseases. In conclusion, in situ reprogramming of reactive glial cells into functional neurons may indicate that direct reprogramming is the first step in brain repair, and this process may be used to replace neurons lost because of nerve injury or disease [72]. Cytotherapy has been shown to improve post-stroke dysfunction. However, identifying the most suitable cell type and its source is still a problem that requires further study. In principle, candidate cells should have high levels of plasticity and the ability to produce different types of neurons; additionally, malignant transdifferentiation should be avoided. Recently, endogenous astrocyte reprogramming into neurons has gradually become a method used to restore nerve function in CNS diseases. It is hoped that astrocyte reprogramming into neurons in glial scars can be applied for nerve tissue regeneration. Unlike stem cell transplantation and recruitment of NSCs from neurogenic regions of the adult brain, reactive astrocytes, which are abundant around the lesion, have the ability to be reprogrammed. Moreover, transformation of reactive astrocytes into neurons not only helps to replace the lost neuron population but also helps to create a more suitable environment for neuron growth and synaptic integration. Future research will highlight the potential of in vivo reprogramming of astrocytes to produce different subtypes of neurons and to identify more suitable astrocyte subsets for post-stroke reprogramming [75]. Furthermore, we will explore more transcriptional regulatory networks that can reprogram astrocytes into specific subtypes of neurons, and use related adeno-associated virus (AAV) to deliver Ascl1 or Neurog2 transcription factors, as these factors can induce reactive astrocytes [76]. Neuroglial cells are reprogrammed and induced to regenerate neurons in a mouse stroke model, which helps to find better ways to screen for alternative populations of neurons that are lost after a stroke [77]. The above summary is shown in Table 2.

**Table 2:** Neuroglial cell reprogramming and neurological diseases

Disease Types	Specie	Transcription Factors	Cell Transdifferentiation	References
PD	Mouse	NeuroD1, ASCL1, LMX1A	Astrocyte→Dopaminergic Neuron	[70, 71]
Brain Damage, AD	Mouse	NeuroD1	Astrocyte→Glutamatergic Neuron	[72]
Brain Damage, AD	Mouse	NeuroD1	NG2 glial cell→ Glutamatergic Neuron	[72]
Stroke	Mouse	ASCL1, Neurog2	Reactive astrocyte→ Neuron	[76]

## 6 Prospects

In conclusion, transcription factor-mediated neuroglial reprogramming is a promising field in the study of cell replacement therapy in the nervous system. At present, researchers can directly reprogram astrocytes to form neuronal cells in vivo and in vitro. Compared with in vitro methods, in vivo reprogramming eliminates the process of cell culture and transplantation, which has the benefit of allowing cells to be regulated by transcription factors and other factors that affect cell survival, leading to precise reconstruction of endogenous cells, tissues and organs [45]. It is also worth focusing on the application of chemical reprogramming. Chemicals can reprogram directly from one somatic cell type to another somatic cell type by regulating cell signaling pathways and epigenetic modifications without using transgenes. The production of induced pluripotent stem cells (iPS) often requires the expression of multiple transcription factors mediated by viral vectors, which may disrupt genomic integrity and cellular function. Chemical reprogramming is an ideal way to further reduce the risk of tumorigenesis. To date, many reported research results have shown that the combination of chemicals and cell type-specific media can differentiate somatic cells into desired cell types, including neuronal cells, neuroglial cells, neural stem cells, and brown adipocytes, cardiomyocytes, etc [77]. Zhang [78] believes that small molecules can bind transcription factors and chemically reprogram neuroglial cells into neurons. They added nine small molecule mixtures that inhibited glial cells but activated neuronal signaling pathways in human astrocytes, and successfully reprogrammed astrocytes into neurons within 8-10 days. This chemical reprogramming is mediated through epigenetic regulation and involves the transcriptional activation of NeuroD1 and Neurog2. Finally, it was proved that neurons transformed by human astrocytes can survive in culture for at least 5 months and form a functional synaptic network. Chemically reprogrammed human neurons can survive in mice for more than a month and integrate into local circuits. The study opens a new way to reprogram neuroglial cells into functional neurons using chemicals.

**Figure 1:**
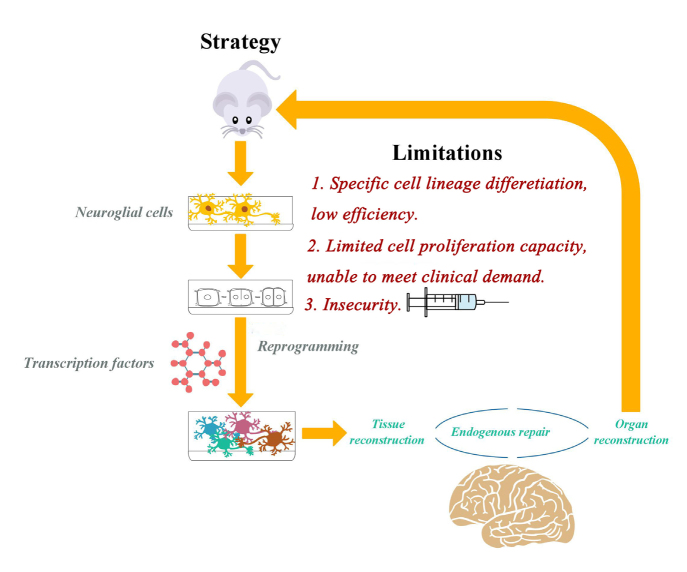
Transcription factor-mediated glial cell reprogramming is a promising area in the study of neuronal cell replacement therapy. Researchers can directly reconstitute astrocytes in and out of rats to form neuronal cells. Thus, endogenous cells, tissues and organs are accurately reconstructed; however, somatic cell reprogramming is dominated by transdifferentiation between specific lineages and limited cell proliferation, low reprogramming efficiency, and genome modification steps still hinder the clinical application of this technology. In order to balance the efficiency of reprogramming and genomic modification, it is necessary to improve existing vectors and explore new vectors and increase their safety and efficiency. [18, 45, 77, 78, 79, 80]

Compared with the CNS, the study of neuroglial cell reprogramming in the PNS is at an early stage. The regeneration ability of the PNS is mainly related to the plasticity of SCs. Therefore, transcription factors involved in myelin formation provide a new therapeutic strategy for cell reprogramming in the case of peripheral nerve diseases or peripheral nerve injuries. Many researchers continue to optimize reprogramming techniques to use stem / progenitor-like glial cells as endogenous cells to repair nerves [18]. Currently, Somatic reprogramming is dominated by transdifferentiation between specific lineages and limited cell proliferation, low reprogramming efficiency and genomic modification steps still inhibit the clinical application of this technology. In order to balance efficiency and genomic modification for reprogramming, current vectors need to be improved and new vectors explored [79]. In the future, we will continue to improve the safety and efficiency of transcription factor-mediated neuroglial cell reprogramming. Cells may be targeted by intravenous injection of safe vectors and transcription factors rather than stereotactic injections [80]. Additional studies are needed to explore different molecular mechanisms in the nervous system and dig deeper into the role and activation mode of reprogramming, which have profound significance for the process of neuroglial cell transdifferentiation (Figure 1).

## Abbreviations

AAV: Adeno-Associated Virus
AD: Alzheimer’s Disease
CNS: Central Nervous System
HDAC1/2: Histone Deacetylases 1 and 2
iANB: induced Adult Neuroblasts
iNSCs: induced Neuronal Stem Cells
iPSCs: induced Pluripotent Stem Cells
MGs: Microglial Cells
NF-κB: Nuclear factor-kappa B
NSCs: Neural Stem Cells
NSLCs: Neuron-like Stem Cells
OPCs: Oligodendrocyte Precursor Cells
PD: Parkinson’s Disease
PKA: Protein Kinase A
PKC: Protein Kinase C
PNS: Peripheral Nervous System
Shh: Sonic hedgehog
TGF-β: Transforming Growth Factor
SCP: Schwann Cell Precursor
SCs: Schwann Cells
